# 

**Published:** 1859-07

**Authors:** 


### CONTENTS OF VOL. XIII.

American Dental Association.......................................................... 623
Another Dental Patent. By W. M. Hunter.......................................... 23
A suggestion.................................................................................... 31
Amalgam Work—Dr. Hunter............................................................. 50
A Proposition.................................................................................. 52
Alveolar Hemorrhage. By George Watt............................................ 57
A Squib............................................................................................ 61
A few ideas on Mechanical Dentistry. By F. J. S. Gorges, D. D. S.......... 77
Artificial Production of Bone.......................................................... 80
A National Society........................................................................... 95
Amalgam Work................................................................................ 108
A Filtering Blow Pipe...................................................................... 114
A Question–Protrusion................................................................... 198
A New Work.................................................................................... 200
An Alarming Case of Hemorrhage, from the Extraction of Two Molar Teeth. By W. H. Shadoan.......... 219
A List of the Alumni of the Ohio College of Dental Surgery, from its organization to the present time.......... 230
A practice to be Corrected.............................................................. 244
A "Crowner's" Quest...................................................................... 245
A Case of Alveolar Abscess............................................................. 248
Amber Bases For Artificial Teeth.................................................... 290
Another Local Society..................................................................... 294
A Practical Treatise on Operative Dentistry..................................... 295
Adaptation of the Human Eye to Distances...................................... 324
Another Screw Loose....................................................................... 352
A Case of Osseous Tumor, with a Tooth Imbedded in the Part............ 358
A Letter from Philadelphia.............................................................. 380
A Correction.................................................................................... 388
A New Society................................................................................. 452
Aromatic Tincture of Myrrh............................................................ 453
An Address at the Commencement of the Dental College of Ohio. By B. P. Aydelott, M. D., D. D. S.......... 462
American Dental Convention—Order of Business.............................. 612
Back Numbers................................................................................. 508
Chloride of Zinc. By J. Taft............................................................. 9
Correspondence............................................................................... 29
Cheap Dentistry and its Tendencies. By Prof. R. Allen, N. Y.............. 65
Correspondence............................................................................... 659, 91
Changes........................................................................................... 98
Conservatory Journal—Devoted to Establishing a Massachusetts Conservatory of Art, Science and Historical Relics.......... 100
Crystal Tin....................................................................................... 150
Crystal Gold..................................................................................... 199
Correspondence............................................................................... 223, 283, 318
Case of Removal of the Lower Jaw. By J. Mason Warren, M. D.......... 326
Cases of Extirpation of the Parotid Gland and other Glandular Tumors.......... 335
Constitution and By-Laws of the Cincinnati Dental Association.......... 377
Case of Sensitive Dentine................................................................. 10

Curiosities ........ 382
Correspondence........ 447
Crystal Gold........ 451
Cincinnati Dental Society........ 507
Case of Arsenical Poisoning. Reported by G. S. Touke........ 580
Cleanliness a Preservative of the Teeth........ 582
Commencement of the Penn. College of Dental Surgeons........ 583
Concave Coundrum Wheels........ 616
Dentist's Assistant. By J. Taft........ 19
Dental Advertising *versus* Dental Ethics. By C. C. Dills........ 20
Delegates of the Mississippi Valley Association........ 27
Delegates of the Ohio Dental College Association........ 28
Discoloration of Gold Fillings........ 28
Dental Depot—Sutton & Raynor........ 52
Dental Education. By J. Taft........ 53
Death From Chloroform........ 72
Dr. Sankester on Food........ 79
Dental Puffing........ 111
Dental Quackery........ 160
Dental Fees. By B. Wood, M. D., Dentist........ 183
Dental Periodical Literature, "Dental Journals"........ 251
Decision of the "Hard Rubber," Contest........ 295
Dental Case........ 315
Declaration of Independence........ 341
Difficult Dentition........ 357
Discussions of the Indiana State Dental Association........ 374
Dental Engagement Book........ 392
Discussions of Michigan State Dental Association........ 429
Dental Patents........ 661
Errata........ 100
Experience with Atmospheric Plates. By A. Berry, D. D. S........ 104
Exchanges—Cool........ 151
Errata........ 242
Essay on Crown Cavities in Molar Teeth. By Geo. L. Paine........ 297
Exposed Nerves. By Ezra McKown........ 332
Elements of Somatology—A Treatise on the General Properties of Matter. By George McIntosh McLhan, M. D........ 451
Excavators and Pluggers........ 451
Fang Filling. By W. H. Shadoan........ 117
Fang Filling or no Fang Filling........ 148
Forgetfulness........ 152
Fang Filling........ 238
Four Sets of Incisors........ 247
Finishing Plate Work. By I. Suesseret........ 330
Fees for Professional Services........ [illegible]
False Teeth in Œsophagus Fifteen Days........ [illegible]
Gold—No. 5........ 43
Gold—No. 6. By Prof. Austin........ 66
Hemorrhage After the Extraction of Three Molar Teeth........ 86
Hunter on Slayton........ 274
Impressions........ 110
Introductory Address. By C. B. Chapman........ 259
Indiana State Dental Association........ 286
Impressions. By Dr. M. M. Oldham........ 305
Indiana State Dental Association........ 359
Irregularity—Treatment. By Dr. George Lupton........ 476
Is there Circulation in the Dentine. By E. Collins........ 596

Little Miami Railroad........ 508
Mood........ 98
Medical and Literary Weekly........ 99
Michigan Dental Association........ 285
Meriam's Tongue Holder........ 323
Meeting of the Michigan Dental Association........ 374
Micrometic, giving without Calculation the Dimensions of Microscopic objects........ 384
Modes of Practice and their Vicissitudes. By J. Taft........ 591
Mad River Valley Dental Association........ 605
Neuralgia Facia........ 81
National Society........ 175
N. B. Slayton........ 189
New York Academy of Medicine........ 440
Nomenclature of Dental Caries........ 448
Ohio College of Dental Surgery........ 199
Os-Artificial........ 505
Pray For Me........ 399
Proceedings of Societies—Cincinnati Dental Association........ 26
Poisoning by Chloroform........ 83
Plastic Filling........ 89
Pressure Plate. By J. Taft........ 112
Proceedings of Societies........ 120
Professional Intercourse........ 153
Popular Instruction........ 243
Professional Health........ 253
Physiology and Pathology of the Nervous System. By Dr. Brown Sequard........ 287
Professional Advertising. By C. C. Dills........ 308
Proceedings of Penn. Society of Dental Surgeons........ 338
"Practical Hint's........ 503
Pathology of the Mouth........ 569
Preservation of the Teeth........ 385
Preparation and Falling of a Simple Cavity in the Right Superior Cuspid, Posterior Proximal Surface. By Dr. J. H. Paine........ 597
Popular Instruction........ 600
Proceeeings and Discussions of the Cincinnati Dental Association........ 602
Plaster Impressions. By Dr. S. Clepenger........ 628
Pathology of the Mouth—Excessive Formation of Epethelium........ 637
Perfect Fillings........ 640
Pivot Teeth. By A. Berry, D. D. S........ 648
Proceedings of Ky. State Dental Association........ 652
Remark on Wisdom Teeth. By Abe O' Robertson, D. D. S., M. D........ 13
Recent Dental Patents........ 91
Remarks on Porcelain Work. By M. Loomis........ 115
Report of Discussions........ 149
Random Thoughts. By George Watt........ 172
Remarks of Prof. McQuillen on Nitric Acid Treatment of Exposed Pulps. By J. Taft........ 194
Reply. By L. H. Hendrick, M. D., D. D. S........ 425
Successful Dental Operations........ 644
Selections........ 31
Stomatitis Materni. By David Prince, M. D........ 87
Soldering Mode Easy........ 181
Superior Stone........ 198
Saint John's Adjustable Lathe........ 235
Selections........ 239
Submucous Injection as a cure for the Tooth-ache of Pregnancy. By H. R. Storer, M.D. 239
Supernumery Teeth........ 248
cientific Artisan........ 248

Selections.......... 287
Sanders' Compound.......... 294
Serum in Pulp Cavities.......... 717
Selections.......... 324
Separating Teeth—By Mechanical Pressure.......... 328
Subjects for Discussions at the next Meeting of the Mississippi Valley Association.......... 383
Superficial Decay. By Geo. S. Fouke.......... 393
Scientific American.......... 453
Sixteenth Annual Meeting of the Miss. Valley Association of Dental Surgeons.......... 479
Sixteenth Annual Meeting of the Miss. Valley Association—Discussions.......... 486
Scientific Artisan.......... 506
Selections.......... 607
Subjects for Discussion by the Western Dental Society, at its next Meeting.......... 611
Sensations attending Artificial Dentures.......... 614
To My Mallet—Apostrophe.......... 650
Thoughts on Fang Filling. By Geo. Watt.......... 5
Treatment of Gold Fillings. By L. C. Whiting.......... 12
The Trip to Niagara.......... 51
The American Journal.......... 50
To Correspondents and Contributors.......... 49
The Meeting at Niagara.......... 48
Treatment of Recently Exposed Nerves with Nitric Acid—with losses. By J. Taft.......... 63
The Lucifer Match Disease, or Rotting of the Jaw Bone.......... 74
The Dental News Letter.......... 97
The Formation of a National Society.......... 147
The "Cosmos".......... 149
The Trip to Niagara.......... 150
The Gold Welding Question.......... 197
The File.......... 215
Thoughts on Fang Filling. By Geo. Watt.......... 220
The Method of putting up the Vulcanite Base for Dentures.......... 236
The D. M. C.'s Reply to "One of her Boys".......... 256
Thoughts for Dentists. By Dr. W. A. Pease.......... 277
The Physiology and Pathology of the Mouth.......... 300
Thoughts for Dentists.......... 318
The Human Teeth.......... 320
The Physiology and Pathology of the Mouth—The Glands and their Secretions.......... 345
The Commencement Exercises.......... 392
The Dentist's Shooting Match.......... 392
Teeth.......... 391
The Physiology and Pathology of the Mouth.......... 457
Thoughts on Decay and its Treatment. By George S. Fouke.......... 576
That "Declaration of Independence".......... 613
Thoughts on Fang Filling. By George Watt.......... 617
The Mallet. By J. Taft.......... 632
Use of Collodian in Exposure of Nerves. By A. Robertson.......... 101
Unusual Protrusion of the Inferior Maxilla.......... 614
Who is Right.......... 99
When and Under what Circumstances should Teeth be Extracted, to secure a Correct Development of the Permanent Teeth? By James Taylor, M. D., D. D. S.......... 201
Warranting Dental Operations.......... 340
Webster's Unabridged Dictionary.......... 616
Vulcanized Base for Teeth—Dr. Roberts, N. Y.......... 51
Vulcanized Rubber.......... 343
Valedictory Address.......... 409

☞ Electro-Magnetism, in the extraction of the teeth, still proves well. I use it quite as frequently, and am better pleased if possible, than hitherto.

T.

☞ We publish the circular of the Corresponding Secretary of the American Dental Convention. If any have failed to receive it direct, they are hereby notified and invited as below:

Augusta, Ga., June 1, 1859.

Dear Sir: "*The American Dental Convention*" will hold its Fifth Annual Session at Niagara Falls, commencing no Tuesday, the second day of August next, at 10 o'clock a. m. At this you are respectfully and cordially invited to be present.

As every faithful and true dentist must feel a deep interest in the advancement of his profession, and an earnest desire that it shall stand second in rank to none of the learned professions, it is expected that each one will encourage every movement calculated to enlighten and improve the great body of "practicing dentists" throughout the country. And, believing that the various "Dental Societies," and especially the "*American Dental Convention*" will do much toward elevating the profession, and thereby prove a powerful auxilliary in attaining the desired object, it is hoped that you will cordially and earnestly coöperate with us.

Yours, Very Respectfully,

D. S. Chase, *Cor. Sec'y.*

